# The Natural History of Inflammatory Bowel Disease in Adults With Common Variable Immunodeficiency: A Case Series From a Single US Tertiary Care Center

**DOI:** 10.1093/crocol/otae062

**Published:** 2025-01-24

**Authors:** Pankhuri Jha, Abbinaya Elangovan, Akash Keluth Chavan, Gregory Cooper, Jeffry Katz, Fabio Cominelli, Miguel Regueiro, Emad Mansoor

**Affiliations:** Department of Medicine, Case Western Reserve University/University Hospitals Cleveland Medical Center, Cleveland, OH, USA; Digestive Health Institute, Case Western Reserve University/University Hospitals Cleveland Medical Center, Cleveland, OH, USA; Department of Medicine, Case Western Reserve University/University Hospitals Cleveland Medical Center, Cleveland, OH, USA; Digestive Health Institute, Case Western Reserve University/University Hospitals Cleveland Medical Center, Cleveland, OH, USA; Digestive Health Institute, Case Western Reserve University/University Hospitals Cleveland Medical Center, Cleveland, OH, USA; Digestive Health Institute, Case Western Reserve University/University Hospitals Cleveland Medical Center, Cleveland, OH, USA; Department of Gastroenterology, Hepatology, and Nutrition, Digestive Disease and Surgery Institute, Cleveland Clinic Foundation, Cleveland, OH, USA; Digestive Health Institute, Case Western Reserve University/University Hospitals Cleveland Medical Center, Cleveland, OH, USA

**Keywords:** common variable immunodeficiency, inflammatory bowel disease, Crohn’s disease, ulcerative colitis

## Abstract

**Background:**

Common variable immunodeficiency (CVID) predisposes patients to inflammatory bowel disease (IBD). There are limited studies characterizing adults with concomitant CVID and IBD (CVID–IBD).

**Methods:**

Demographics, clinical courses, endoscopic findings, and disease-specific therapies were obtained in adults ≥18 years with CVID–IBD over a 5-year period.

**Results:**

We identified 11 patients with CVID–IBD, 6 with Crohn’s disease (CD), and 5 with ulcerative colitis (UC). The presenting symptoms in 10 (91%) were abdominal pain and diarrhea. A majority of patients were diagnosed with IBD before CVID. Patients with CVID–IBD were diagnosed with CVID at an older age compared to patients with CVID only. The most prevalent endoscopic finding in CD was either erythema or erosions in the ileum, whereas in UC, it was pancolitis. One patient with CD and 1 with UC required treatment with immunomodulators to achieve remission. Three patients with CD and 2 with UC required treatment with biologics to achieve remission. Eight received intravenous immunoglobulin (IVIG) for CVID. Four patients with CD and 1 with UC were diagnosed with either leukemia or lymphoma, and the most common malignancy was chronic lymphocytic leukemia (*n* = 3).

**Conclusions:**

This is one of few studies evaluating demographic, clinical, endoscopic, histologic features, and therapeutic management in patients with CVID–IBD. Further studies are required to elucidate long-term differences in the clinical course and treatment of patients with CVID–IBD.

## Graphical Abstract

**Graphical Abstract:**
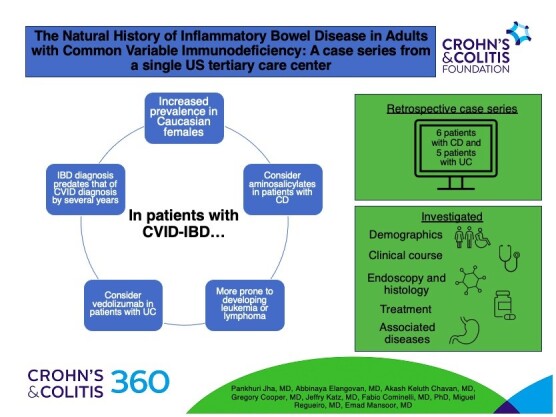

**Key Points**

- Patients with inflammatory bowel disease (IBD) may develop a diagnosis of common variable immunodeficiency (CVID) several years after their initial IBD diagnosis.
- Patients with CVID–IBD are more prone to developing leukemia or lymphoma, although patients with IBD alone are not thought to have a similar increased risk.
- Providers can consider biologic therapy in patients with CVID–IBD to achieve disease remission, however, they should weigh the risks and benefits of such advanced therapies given the high risk of infections and malignancies in this subpopulation.

## Introduction

Common variable immunodeficiency (CVID) is the most common symptomatic primary immunodeficiency (PID).^1^ Its pathophysiology involves impaired B-cell differentiation that leads to defective immunoglobulin production, thus predisposing patients to recurrent infections and inflammatory disorders including inflammatory bowel disease (IBD).^2^ The prevalence of CVID in the general population is estimated to range between 1/50 000 and 1/200 000.^3^ The prevalence of CVID is thought to be higher in Caucasians, between 1/50 000 and 1/125 000, with equal prevalence between males and females.^4^ Few studies have explored the prevalence of IBD in CVID specifically, and those performed have focused on pediatric populations. There is otherwise no published literature describing the epidemiology of concomitant CVID and IBD (CVID–IBD) in adults.

Gastrointestinal (GI) manifestations including infectious diarrhea are common in CVID, thus making the diagnosis of IBD in CVID challenging. Endoscopy findings in patients with CVID and GI symptoms are nonspecific, with atrophic and chronic gastritis and colon ulcerations being commonly described.^5,6^ Histopathology typically reveals a variety of findings including cryptitis, granulomas, neutrophilic and lymphocytic infiltrates, nodular lymphoid hyperplasia, reduced plasma cells, increased intraepithelial lymphocytes (IEL), and duodenal villous atrophy.^2,5–8^ While an absence of plasma cells can suggest GI tract involvement in CVID, this finding is only present in 66% of patients.^8^ Hence, the diagnosis cannot always be made in isolation and without other supportive findings.

Making an accurate diagnosis is important, as patients with CVID–IBD require individualized treatment strategies that are separate from patients with CVID alone. For example, while intravenous immunoglobulin (IVIG) normally improves the infectious complications of CVID, the same effect has not been noted for either inflammatory, including IBD, or autoimmune manifestations of the disease.^9,10^ We found 1 study describing a treatment regimen for patients with IBD and PIDs, consisting of corticosteroids and immunomodulators, followed by biologics in the setting of treatment failure.^5^ However, this study did not further delineate IBD-specific therapies in patients with Crohn’s disease (CD) compared to patients with ulcerative colitis (UC).

Numerous studies have characterized demographics, clinical presentations, and endoscopic and pathologic findings in patients with CVID and GI symptoms. One study has also described treatment regimens in patients with concomitant IBD and PIDs. However, there remains a paucity of data about clinical presentation, diagnosis, and management of patients with CVID–IBD, especially when comparing patients with CD to those with UC. We aim to describe demographics, natural history, clinical presentations, endoscopic and pathologic findings, and therapeutic strategies in adult patients with CVID–IBD to gain a better understanding of how to manage these patients and optimize their treatment regimens.

## Materials and Methods

We performed a retrospective case series in adults ≥18 years with CVID–IBD over a 5-year period (between 2017 and 2022) at University Hospitals Cleveland Medical Center in Cleveland, Ohio. The study was approved by University Hospital’s Institutional Review Board.

We used a national de-identified health research network with data sourced from 84 healthcare organizations (HCO) within the United States (TriNetX). Data was accessed until July 18, 2022. TriNetX does not identify participating HCOs, however, a typical partner HCO includes a large academic health center with inpatient, outpatient, and specialty care services. Data is received directly from participating HCOs, including an extensive data quality and accuracy assessment.

Patients with a diagnosis of CVID (ICD-10: D83.9) were identified per ICD-10-CM coding. A diagnosis of CVID was made based on the International Consensus Document guidelines that list 5 criteria for diagnosis: significantly low serum concentrations of IgG antibodies, low serum concentrations of IgA and/or IgM antibodies, a poor or absent response to immunization, greater than 2 years of age, and absence of any other defined immunodeficiency.^11^ Patients with IBD were defined as individuals carrying a diagnosis of either CD (ICD-10: K50.90) or UC (ICD-10: K51.90) per ICD-10-CM coding and a prescription for at least 1 IBD-specific medication including corticosteroids (budesonide, methylprednisolone, and prednisone), aminosalicylates (balsalazide, mesalamine, and sulfasalazine), immunomodulators (azathioprine, 6-mercaptopurine, and methotrexate), biologics (adalimumab, certolizumab pegol, golimumab, infliximab, risankizumab, ustekinumab, and vedolizumab), and small molecules (ozanimod, tofacitinib, and upadacitinib). The diagnosis of IBD could have been made before or after a diagnosis of CVID. Only patients with concomitant diagnoses of CVID and IBD were included in the study.

A subsequent manual chart review of the electronic health record was performed by 2 independent reviewers (P.J. and A.E.) to confirm the diagnosis of CVID–IBD, and cases with agreement between both reviewers were included. In cases of disagreement, a third reviewer (E.M.) evaluated cases for resolution. We collected data on demographics, dates of CVID and IBD diagnoses, sequence of CVID and IBD diagnoses, initial presenting symptoms, objective markers of inflammation including C-reactive protein (CRP) and fecal calprotectin at the patient’s index visit, endoscopic findings on esophagogastroduodenoscopy (EGD) and colonoscopy following index visit, histopathology, disease-specific therapies for CVID and IBD, and prevalence of other associated diseases including GI and respiratory infections, leukemia, lymphoma, and Celiac disease. The index visit was defined as the first appointment at which a patient established with a gastroenterologist for their IBD care.

Patients were additionally evaluated for gene mutations known to mimic IBD with immunodeficiency and were consequently excluded if found to have insufficiency of the following genes: cytotoxic T-lymphocyte-associated-antigen 4 (CTLA-4), lipopolysaccharide-responsive beige-like anchor protein (LRBA), and interleukin-21 (IL-21).^12–14^ As markers of chronic inflammation, we selected crypt architectural distortion, cryptitis, crypt abscesses, diffuse mixed lamina propria inflammation, basal plasmacytosis, and basally located lymphoid aggregates.^6,15^ Pathology reports were reviewed extensively to ensure that these chronic findings of IBD inflammation were present. Patients with either a paucity or absence of plasma cells on histology suggestive of CVID-associated enterocolitis were excluded.^16^ We defined complete remission as evidence of clinical, endoscopic, and histologic remission for the purposes of our study.

## Results

We identified 11 patients with CVID–IBD. Six patients were diagnosed with CD and 5 patients were diagnosed with UC. Seven patients (64%) were female. The mean age at CVID diagnosis was 34 years and the mean age at IBD diagnosis was 30 years. No patients were found to have gene mutations mimicking IBD with immunodeficiency. No patients were found to have a paucity or absence of plasma cells on histology requiring exclusion.

Two patients (18%) across both cohorts required immunomodulators after IBD diagnosis. Both patients treated with either 6-mercaptoupurine (*n* = 1) or azathioprine (*n* = 1) had improvement in clinical symptoms. Both patients developed endoscopic and histologic remission after initiation of an immunomodulator.

Five patients (45%) across both cohorts required advanced therapies with biologics after IBD diagnosis. All patients treated with ustekinumab (*n* = 1), vedolizumab (*n* = 3), or certolizumab (*n* = 1) had improvement in clinical symptoms. Follow-up endoscopy and histology were available for 4 patients. Three patients (60%) developed endoscopic and histologic remission after initiation of a biologic. One patient (20%) had evidence of quiescent disease with chronic focal active colitis on histology.

### Crohn’s Disease and CVID

#### Demographics and clinical features

Among the 6 patients with CD, the mean age at CVID diagnosis was 34 years and the mean age at CD diagnosis was 25 years (Table 1). Four patients (67%) were Caucasian females. Five patients (83%) presented with either abdominal pain or non-bloody diarrhea as the initial symptom of CD (Table 2). One patient did not present with either of these and instead had rectal pain as the presenting symptom of his CD. At the index visit, elevated inflammatory markers were noted in 4 patients (mean CRP 7.8 [*n* = 4] and mean fecal calprotectin 204.5 [*n* = 2]). CRP and fecal calprotectin data were not found for the 2 patients.

**Table 1.** Demographic features of patients with concomitant CVID and IBD.

Patient	IBD type	Sex	Race	Age at CVID diagnosis	Age at IBD diagnosis
1	CD	F	Caucasian	7	20
2	UC	M	Caucasian	68	48
3	UC	F	Caucasian	53	30
4	CD	F	Caucasian	45	13
5	CD	M	Black	36	37
6	UC	F	Caucasian	15	26
7	UC	F	Caucasian	62	40
8	CD	F	Caucasian	35	26
9	CD	F	Caucasian	39	4
10	UC	M	Caucasian	35	18
11	CD	M	Caucasian	43	48

Abbreviations: CD, Crohn’s disease; CVID, common variable immunodeficiency; IBD, inflammatory bowel disease; UC, ulcerative colitis.

**Table 2.** Presenting symptoms at diagnosis, Montreal classification at diagnosis, endoscopic, and histologic findings after index visit of patients with concomitant CVID and IBD.

Patient	IBD type	Presenting IBD symptoms	Montreal classification at diagnosis	Endoscopy macroscopic findings after index visit	Endoscopy histologic findings after index visit	Colonoscopy macroscopic findings after index visit	Colonoscopy histology findings after index visit
1	CD	Non-bloody diarrhea & weight loss	A2, L3, and B1	Normal	Normal	Pancolitis, ileitis	Granulomas in TI
2	UC	Bloody diarrhea	E3	Erythema in stomach	Normal	Active pancolitis	Focal active cryptitis without granuloma or crypt abscess, chronic inflammatory colitis in lamina propria
3	UC	Abdominal pain, bloody diarrhea, tenesmus	E3	Erythema and friability in the gastric antrum and body	Chronic inactive gastritis	Normal	Normal
4	CD	Rectal pain and perianal fistula	A1, L1, B1	Erythema in neo-TI	Gastritis, focal enteritis, focal colitis without granuloma	Erythema, erosions in neo-TI	Focal active colitis at ileocecal anastomosis without granuloma
5	CD	Right-sided abdominal pain, rectal pain, perianal fistula and non-bloody diarrhea	A2, L2, and B3	Erythema in gastric body and antrum	Gastritis, increased intraepithelial lymphocytes with preserved villous architecture and TI	Normal	Patchy acute colitis
6	UC	Left-sided abdominal pain and bloody diarrhea	E3	Normal	Normal	Pancolitis, erosive, erythematous, friable, and granular mucosa	Focal active cryptitis with architectural distortion, crypt abscess without granuloma, and basal plasmacytosis
7	UC	Bloody diarrhea	E3	Not performed	Not performed	Pancolitis, congested, erythematous, friable, and granular mucosa	Chronic colitis with crypt branching and without crypt abscess or cryptitis
8	CD	Abdominal pain and non-bloody diarrhea	A2, L3, and B1	Erythema in gastric body, antrum, and duodenum	Normal	Ileocecal erythema and rectal inflammation	Focal active cryptitis and crypt abscess without granuloma
9	CD	Abdominal pain and bloody diarrhea	A1, L1, and B1	Erythema in the antrum and pre-pylorus	Gastritis	Ileal erythema and scattered aphthous ulcers	Chronic active ileitis without granuloma or crypt abscess
10	UC	Abdominal pain	E1	Erythema in gastric body, antrum, and duodenum	Gastritis	Rectal erythema	Normal
11	CD	Non-bloody diarrhea & weight loss	A3, L1, and B1	Normal	Normal	Ulcerated mucosa at ileocecal valve and erythematous and nodular mucosa in TI	Focal active ileitis and chronic enteritis at ileocecal valve

Abbreviations: CD, Crohn’s disease; CVID, common variable immunodeficiency; IBD, inflammatory bowel disease; TI, terminal ileum; UC, ulcerative colitis.

Montreal classification for Crohn’s disease:

Age at diagnosis (years): A1, ≤16; A2, 17-40; A3, >40.

Location: L1, ileal; L2, colonic; L3, ileocolonic.

Behavior: B1, non-stricturing, non-penetrating; B2, stricturing; B3, penetrating.

Montreal classification for ulcerative colitis:

Extent: E1, ulcerative proctitis; E2, left-sided UC (distal UC); E3, extensive UC (pancolitis).

#### Endoscopy and histology findings

During the EGD following index visit, 4 patients (67%) had erythematous mucosa in the gastric body, antrum, or ileum (Table 2). The most prevalent histology findings were gastritis in 3 patients (50%), with increased intraepithelial lymphocytes (IEL) noted in 1 patient. Two patients (33%) had normal macroscopic and histologic findings on EGD. Five patients (83%) had erythema or erosions in the ileum on colonoscopy. Corresponding histology included ileitis in 3 patients (50%), and cryptitis or granulomas in 2 patients (33%). At initial CD diagnosis, 1 had only colonic involvement, 3 had only ileal involvement, and 2 had both colonic and ileal involvement (Table 2).

#### Treatment

Four patients (67%) were initiated on aminosalicylates and 2 patients achieved complete remission with its use (Table 3). One patient subsequently required escalation to immunomodulator therapy that helped achieve complete remission. One patient required advanced therapy with adalimumab and later ustekinumab. The decision to treat with ustekinumab was made after the patient developed an adverse reaction to adalimumab. This patient had improvement in clinical symptoms with ustekinumab. Follow-up endoscopy reports were unavailable to assess for consequent endoscopic and histologic remission.

**Table 3.** Prior IBD therapy, therapy inducing remission, and relevant surgeries of patients with concomitant CVID and IBD.

Patient	IBD type	Prior IBD therapy	Therapy inducing remission	Surgeries
1	CD	Mesalamine and adalimumab	Ustekinumab	None
2	UC	Prednisone	Prednisone	None
3	UC	Sulfasalazine and mesalamine	Mesalamine	None
4	CD	Mesalamine, azathioprine, and 6-mercaptopurine	6-Mercaptopurine	Small bowel resections, right hemicolectomy with ileocolonic anastomosis, and stricturoplasty
5	CD	Vedolizumab	Vedolizumab	None
6	UC	Mesalamine and vedolizumab	Vedolizumab	None
7	UC	Sulfasalazine, mesalamine, and vedolizumab	Vedolizumab	None
8	CD	Balsalazide	Balsalazide	None
9	CD	6-Mercaptopurine, adalimumab, and infliximab certolizumab	Certolizumab	Colectomy with ileoanal anastomosis and completion proctectomy with end ileostomy
10	UC	Azathioprine	Azathioprine	None
11	CD	Mesalamine	Mesalamine	None

Abbreviations: CD, Crohn’s disease; CVID, common variable immunodeficiency; IBD, inflammatory bowel disease; UC, ulcerative colitis.

One patient (17%), on chronic prednisone for unclear reasons, was initiated on immunomodulator therapy (Table 3). However, the patient developed agranulocytosis and thus required treatment with advanced therapies. The patient was trialed on adalimumab and infliximab but developed antibodies to both. Thus, the patient was switched to certolizumab which helped achieve complete remission.

One patient (17%), only with colonic involvement and recurrent perianal fistulas, was initiated on vedolizumab (Table 3). The decision to use this agent was due to this patient’s history of lymphoma, which limited the use of immunomodulators, anti-TNF medications, and ustekinumab. All 4 patients with CD requiring either immunomodulators or advanced therapies had either improved clinical symptoms and/or endoscopic and histologic remission.

Five patients (83%) received supplementary IVIG every 2-4 weeks. Unfortunately, there was no documentation explaining why 1 patient did not receive IVIG. Two patients (33%) underwent surgeries, and both were colectomies with either an ileocolonic anastomosis or ileoanal anastomosis (Table 3).

### Ulcerative colitis and CVID

#### Demographics and clinical features

Among the 5 patients with UC, the mean age at CVID diagnosis was 47 years and the mean age at UC diagnosis was 32 years (Table 1). Three patients (60%) were Caucasian females. All patients presented with either abdominal pain or bloody diarrhea as the initial symptom of UC (Table 2). At the index visit, elevated inflammatory markers were noted in 4 patients (mean CRP 53 [*n* = 4] and mean fecal calprotectin 100 [*n* = 3]). CRP and fecal calprotectin data were not found for 1 patient.

#### Endoscopy and histology findings

Three patients (60%) had pancolitis on colonoscopy (Table 2). Corresponding histology included focal active cryptitis in 3 patients (60%). Crypt abscesses and architectural distortion were found in 2 patients (20%).

#### Treatment

Three patients (60%) were initiated on aminosalicylates, and 1 patient achieved complete remission with its use (Table 3). The other 2 patients were escalated to advanced therapies, notably vedolizumab. One patient was escalated after developing sulfasalazine-induced pancytopenia. After initiation of vedolizumab, the patient had significant clinical improvement, and follow-up endoscopy was with evidence of quiescent disease with chronic focal active colitis on histology. The other patient was escalated due to clinical relapse. This patient developed complete remission.

One patient (20%) was initiated on azathioprine with complete remission (Table 3). One patient required no maintenance therapy and was treated with only prednisone tapers for his disease flare-ups. All 3 patients with UC requiring either immunomodulators or advanced therapies had either improved clinical symptoms and/or endoscopic and histologic remission.

Three patients (60%) received supplementary IVIG every 2-4 weeks. The remaining 2 patients were recommended to start IVIG, however both were lost to follow-up.

### Prevalence of Other CVID-Associated Diseases

*Clostridium difficile* was the most prevalent pathogen implicated in GI infections, found in 4 of 11 total patients (36%). Sixty-six percent of patients treated with vedolizumab were more likely to develop *C. difficile* infection compared to 25% of patients not treated with vedolizumab. Ten patients (91%) had prior respiratory infections, however, only 2 patients (18%) developed bronchiectasis. All 7 patients (100%) treated with either immunomodulators or advanced therapies had increased rates of both GI and respiratory infections. An increased infection rate was also found among patients not on these therapies.

Just under half of our cohort (45%) were diagnosed with either leukemia or lymphoma. The most prevalent malignancy diagnosed was chronic lymphocytic leukemia (CLL, *n* = 3). Other malignancies found include non-Hodgkin lymphoma (NHL, *n* = 2) and follicular lymphoma (*n* = 1). One patient was diagnosed with both CLL and NHL. Three patients (27%) were diagnosed with either leukemia or lymphoma prior to their CVID diagnosis. Among patients with CLL, 1 patient (20%) was treated with rituximab, 1 patient was treated with intrathecal methotrexate, and 1 patient did not receive treatment.

Six patients across both cohorts (55%) were tested for Celiac disease; however, testing was negative in all patients. Vitamin B12 levels were tested in 9 patients (82%) but were found to be normal in all patients as well.

## Discussion

To our knowledge, this is one of the few studies dedicated to characterizing demographics, clinical courses, endoscopic, histologic features, disease-specific therapies, and other diseases in patients with concomitant CVID and IBD, specifically comparing findings between patients with CD compared with UC.

Among patients with IBD, females have a higher risk of developing CD after puberty, and males have a higher risk of developing UC after 45 years.^17^ We found patients with CVID–IBD to be predominantly Caucasian females, suggesting an increased female prevalence in patients with concomitant CVID compared to those with IBD alone. This is consistent with prior studies that have characterized patients with CVID and GI symptoms.^2^

We found most patients were diagnosed with IBD first followed by CVID. Literature describing patients with CVID and GI symptoms demonstrate the opposite, that a diagnosis of CVID predates that of GI symptom onset.^2^ However, this study did not specifically characterize patients with IBD. Perhaps our findings may be explained by an unknown intracellular mechanism, wherein the abnormal T-cell mediated response thought to drive IBD interacts with B cells in a way that causes reduced immunoglobulin production, thus predisposing patients to CVID. We also acknowledge the discrepancy in our findings may be related to our overall smaller sample size. More research needs to be done in eliciting the temporal relationship between CVID and IBD specifically.

On average, patients with UC were diagnosed with CVID at an older age, 47 years, compared to patients with CD, 34 years. Notably, studies report the average age for CVID disease onset is 26 years.^18^ We report that in patients with underlying IBD, a diagnosis of CVID can be found up to 20 years after the average age of CVID diagnosis. The etiology for this is not clear. However, our findings should alert providers to monitor for future diagnoses of CVID in patients with IBD, especially if patients present with increased sinopulmonary infections.^1^

The presenting IBD symptoms in 10 patients (91%) were abdominal pain and diarrhea. A majority had elevated inflammatory markers including CRP and fecal calprotectin, which has previously been noted in patients with CVID and GI symptoms.^6^ We propose these symptoms and elevated markers should assist providers in diagnosing patients with CVID–IBD.

We found patients with concomitant CD were likely to have erythematous mucosa on upper endoscopy with corresponding gastritis on histology. On colonoscopy, ileal erosions or erythema was the most common finding with evidence of ileitis, cryptitis, and granulomas on pathology. We also found patients with concomitant UC were likely to have pancolitis on colonoscopy with corresponding cryptitis, crypt abscesses, and architectural distortion on histology. These findings are like those outlined in prior studies when describing GI disease in patients with CVID.^4^ If there is not a paucity or absence of plasma cells on histology, as there was in our study, providers should feel more confident diagnosing these patients with CD or UC, as opposed to CVID involvement of the GI tract.

Despite not often being utilized in CD, half of the patients initiated on aminosalicylates achieved complete remission of their disease. Thus, providers may consider its use in patients with concomitant CVID and CD. We found that patients on biologics had the highest success of achieving complete remission of their CD. Thus, if patients are refractory to initial therapies, providers can consider utilizing biologics in patients with CVID and concomitant CD, as is typically the case in patients with CD alone. On the contrary, although aminosalicylates are considered first-line therapy in patients with mild UC, not all our patients initially achieved remission with its use. We found patients developed improved clinical symptoms, with one achieving complete remission, with the use of vedolizumab. The success of vedolizumab has been noted in patients with CVID and gut inflammation, however, its role in patients with concomitant CVID and UC specifically has not yet been described.^19^ We propose that providers consider escalating to non-TNF-alpha biologics, including vedolizumab, in patients with concomitant CVID and UC. However, we caution that providers should closely weigh the risks and benefits of such advanced therapies, especially given the higher risk of infections and malignancies in this subpopulation that we found.

The most common etiology of GI infections in our cohort was *C. difficile*, even though the literature purports *Giardia lamblia* as the most common GI infection to afflict patients with CVID.^20^ We also found increased rates of respiratory infections in almost our entire cohort. However, our data also shows an increased number of GI and respiratory infections in patients on immunomodulators and advanced therapies. Thus, whether the rates of infections and type of bacteria causing the infections are due to the inherent nature of CVID or specific therapies warrants further investigation.

Notably, we found that just under half of our patient cohort (45%) were diagnosed with either leukemia or lymphoma. It is well-known that patients with CVID are at an increased risk for developing lymphomas, especially in a background of nodular lymphoid hyperplasia.^7,21^ The prevalence of lymphoma in patients with CVID appears to range between 1.4% and 4.1%.^21,22^ An increased incidence of leukemia has also been noted in patients with CVID, though at considerably less rates compared with lymphoma. For example, 1 study mentions only 0.6% of their 1091 patient cohort with CVID as having leukemia.^23^ Another study has demonstrated that 2%-10% of patients with CVID are at increased risk for developing lymphoid malignancies, usually non-Hodgkin’s B-cell lymphoma with GI tract involvement.^4^ Furthermore, a study of GI pathology in patients with CVID revealed small bowel lymphoma occurring in 2/43 patients (4.6%) with CVID.^21^ Interestingly, of our 2 patients diagnosed with NHL, neither had GI tract involvement. Instead, the NHL involved the lung and maxillary sinus.

To our knowledge, there are no studies that have identified the prevalence of leukemia and lymphoma in patients with CVID–IBD specifically. Our study demonstrates an increased prevalence of leukemia, most notably CLL, and lymphoma among patients with CVID–IBD. This is especially interesting, as a recent review noted the incidence of leukemia and lymphoma in patients with IBD alone is low.^24^ Furthermore, given that leukemia and lymphoma can lead to secondary hypogammglobulinemia, one would expect the onset of malignancy to predate a diagnosis of CVID, which our study’s data did support. However, since this is a single-center retrospective study limited by a small sample size and selection bias, these findings need to be confirmed in large prospective studies. Whether the higher prevalence of malignancies in this patient population bears any causation to an underlying co-diagnosis of IBD on top of a known association of malignancy with CVID is an area that certainly requires further study.

Strengths of our study include the methodology, notably our inclusion and exclusion criteria. Although our sample size was small, we only included patients with a clear diagnosis of concomitant CVID and IBD. No patients had underlying gene mutations mimicking IBD with immunodeficiency and we excluded patients if there was concern for CVID-associated enterocolitis. However, we acknowledge the sensitivity of the finding of the paucity or absence of plasma cells on histology used to define CVID-associated enterocolitis is not high, thus representing a limitation of our study. Other limitations otherwise include retrospective analysis that lends itself to missing data.

In conclusion, this is one of the few studies dedicated to characterizing patients with CVID–IBD. Important findings include monitoring for a future CVID diagnosis in patients with established IBD, utilizing vedolizumab to achieve complete remission in patients with CVID and concomitant UC, and anticipating a diagnosis of leukemia or lymphoma in patients with CVID–IBD. We want to emphasize continuing to utilize the dearth or absence of plasma cells in helping make a definitive diagnosis of IBD in patients with CVID. Furthermore, we reiterate that providers can consider utilizing biologic therapy in patients with CVID–IBD but should weigh the risks and benefits given the higher risk of infections and malignancies in this subpopulation. For many patients, GI symptoms may be the first and only clinical presentation of their underlying immunodeficiency. This is not to be taken lightly, as GI complication-related morbidity is only secondary to that of the respiratory tract.^3^ Thus, the classification of patients with concomitant CVID and IBD is imperative.

## Data Availability

The data underlying this article are available in the article and in its online supplementary material.
